# The Story of the Insane from Year to Year

**Published:** 1897-02-06

**Authors:** 


### THE STORY OF THE INSANE FROM YEAR TO YEAR.

Ninth Series.

### THE RETREAT, YORK.

This asylum issued its one-hundredth report, and it opened and closed its year with the same number of patients in residence, namely, 158. There were 44 admissions, 25 recoveries, and 5 deaths. Calculated in the usual manner, the percentage of recoveries was 53·72, and that of the deaths was 3·15. Of course, no useful comparison can be drawn between the recovery and death rate of a county asylum and such an institution as the Retreat, because the class of cases admitted differs very materially. There are in the Retreat 34 patients who pay only 12s. a week, and there are 20 who pay less than 25s., and as the average weekly cost of maintenance is 35s., it is evident that the asylum is doing useful, charitable work. Dr. Pierce thinks it would be a good plan to transfer certain patients from one asylum to another. He refers to discontented cases, and most truly says that, "After many years' residence in one place, they become polarised, as it were, with attractions here and antipathies there, both of which may be far from healthy." We know this type well, and we feel sure the neighbouring county asylums could supply Dr. Pierce with as many as he wants; but whether they would be equally ready to admit his cases is matter of considerable doubt. There was at one time a great outcry against restraint in any form and for any purpose, and we have been threatened with a similar denunciation of seclusion and a tendency to boast of the rarity in which it is resorted to. We are pleased to find that Dr. Pierce writes that "the utility and humaneness of secluding certain violent patients admits, of no doubt in my mind, if done with proper care."

### DEVON COUNTY ASYLUM.

On January 1st, 1895, this asylum contained 1,063 patients, and on December 31st the number had fallen to 1,057. There were 160 admissions, 43 readmissions, 74 recoveries, and 99 deaths. Calculated on the admissions, the recoveries stand at 36·94 per cent,, and the deaths at 9·39 on the average number resident; both of these percentages being close on the average of county asylums. Of the deaths 25 were caused by cerebro-spinal diseases; 20 by consumption; 2 to dysentery; and 2 to enteritis. Of the admissions 34 were driven insane by various moral causes; 9 by intemperance; and 50 by hereditary tendency. Dr. Saunders says that "the ever-increasing demand for accommodation for the insane poor of the county has taxed the resources of the asylum in a way which it would be difficult to exaggerate, and the building of new wards are not likely to be completed in the present century." Why not? we might ask. There are several years of the century to run yet, and the whole asylum might be rebuilt in the time. The old water supply failed, but a new bore-well has been sunk, and the yield of water would seem to be far in excess of the requirements. The weekly cost of maintenance per head was 8s. 6d.

